# Optimal Treatment for Subarachnoid Neurocysticercosis: Closer, but Not There yet

**DOI:** 10.4269/ajtmh.19-0754

**Published:** 2019-10-28

**Authors:** A. Clinton White, Agnes Fleury

**Affiliations:** 1 Infectious Disease Division, Department of Internal Medicine, University of Texas Medical Branch, Galveston, Texas;; 2 Instituto de Investigaciones Biomédicas-UNAM/Instituto Nacional de Neurología y Neurocirugía/Facultad de Medicina-UNAM, Ciudad de México, Mexico

Neurocysticercosis, caused by *Taenia solium*, presents with a spectrum of disease.^1^ The most common presentation in patients diagnosed in India and the United States is with seizures and/or headaches and a single enhancing lesion. Patients with this presentation have a good prognosis. In Latin America, most patients come to medical attention with multiple cystic lesions and recurrent seizures. Both of these groups seem to do well when treated with antiepileptic drugs, steroids, and antiparasitic drugs. Patients with multiple parenchymal cysts may benefit from combination therapy with albendazole and praziquantel.^2^ In endemic villages, population-based surveys note that it is more common to have calcified lesions from prior cystic lesions.^3–5^ Many cases are asymptomatic, but patients may also have chronic epilepsy. A minority of patients present with extra-parenchymal disease with cysticerci in the ventricles causing obstructive hydrocephalus or with subarachnoid disease associated with arachnoiditis.^6^ The latter are associated with a particularly poor prognosis.

In 1987, Sotelo and Marin^7^ published a pivotal article describing the natural history of 92 patients with hydrocephalus due to subarachnoid neurocysticercosis. All of them underwent shunt placement to ameliorate hydrocephalus. Despite surgical intervention, the prognosis remained poor: 34 died within 2 years and only half survived to the last follow-up (median 8 years). Most of the survivors had continued arachnoiditis. In contrast to that report and some more recent reports,^8^ Nash and others^9^ report no fatalities with subarachnoid neurocysticercosis from a series of 30 patients followed at the NIH in the United States. Other series have also noted few fatalities with optimal management.^10,11^ Why has there been such a stark difference in case fatality rates between studies? The main difference in the management of patients between the older series and more recent reports is the recent aggressive use of antiparasitic and anti-inflammatory drugs and aggressive management of increased intracranial pressure.

The role of antiparasitic drugs in neurocysticercosis has been controversial. Early on Sotelo and others noted favorable responses when patients with parenchymal neurocysticercosis were treated with antiparasitic drugs.^12,13^ However, the initial studies were not optimally controlled, and the first randomized trial suggested no benefit.^14^ Subsequent well-designed, double-blind, randomized trials demonstrated that antiparasitic drugs for cystic parenchymal lesions clearly hastened cyst resolution and led to fewer generalized seizures.^15,16^ The effect was more limited for patients with single enhancing lesions, but meta-analyses continue to demonstrate that treatment with albendazole plus corticosteroids led to more rapid radiographic resolution and fewer seizures in the short term.^17^ The role of antiparasitic drugs in extra-parenchymal disease has been even more controversial. For example, Agapejev documented a 36% fatality rate with albendazole treatment.^18^ By contrast, Proaño and others^19^ noted that although patients with giant cysticerci (who mostly had subarachnoid neurocysticercosis) responded poorly to single courses of antiparasitic drugs, most had a good prognosis when treated with repeated courses. Nash and others^9^ took a different approach. Instead, patients were treated with prolonged courses of antiparasitic drugs, typically lasting 6–12 months. This was associated with resolution in all but one case, and that case was still on treatment at the time of writing. Some were treated with a combination of praziquantel and albendazole. Combination therapy could potentially kill parasites more rapidly, and further studies of this approach are needed. Other investigators have used high-dose albendazole in short cycles, repeated as needed, with results similar to those noted by Nash.^6,10^

Clearly, host inflammation plays a key role in the pathogenesis of neurocysticercosis. Nash and others^9^ also used an aggressive approach to anti-inflammatory therapy. Most of the patients received high doses of corticosteroids for over a year. To continue anti-inflammatory therapy in the presence of or in anticipation of steroid-related adverse events, most of the patients were also treated with methotrexate and/or etanercept.^9,20^ However, there was significant variability in regimens between patients. Thus, although anti-inflammatory therapy appears to be critically important, more studies are needed on optimal agents, doses, and duration and on how to individualize therapy.

A third aspect of this case series is the use of neurosurgery. Fourteen patients (41%) required placement of a ventriculoperitoneal shunt and one underwent third ventriculostomy.^9^ Although shunt failure was noted, it was less frequent than in older series. This may have been due to the use of antiparasitic drugs and/or anti-inflammatory treatment, which were associated with improved shunt survival in other series.^21,22^ Others have proposed debulking via minimally invasive neurosurgery.^23^ This was not performed in the series by Nash and others and remains an experimental approach.

If patients are treated with prolonged or repeated courses of antiparasitic drugs, how long should the treatment be continued and what are the end points? Resolution by neuroimaging does not seem to correlate well with long-term response. For example, Nash and others noted three patients who relapsed after apparent radiologic resolution.^9^ In other cases, scarring may lead to an incomplete radiologic response even in the setting of elimination of viable parasites. Nash and others now propose using monoclonal antibody–based, antigen-capture ELISA assays as a way of following the therapeutic response. This has previously been demonstrated to better correlate with the response to therapy than does follow-up imaging.^24^ However, there is currently limited availability of antigen detection assays in the United States. More recently, a real time PCR assay has been proposed for use in the follow-up of patients with subarachnoid neurocysticercosis.^25^ Clearly, further studies are needed to determine optimal duration of therapy and clinical end points.

Neurocysticercosis represents a spectrum of disease with variable clinical presentation and optimal management. In all forms of disease, symptomatic therapy is critically important, including aggressive management of elevated intracranial pressure. Antiparasitic drugs, anti-inflammatory drugs, and neurosurgery all play a key role in management. For subarachnoid neurocysticercosis, the data from Nash and others suggest that aggressive therapy with prolonged courses or high doses of antiparasitic and anti-inflammatory therapy is beneficial.^1^ They also suggest that individualization of management based on antigen detection and inflammatory markers should improve prognoses. Even with this aggressive approach, most patients had at least some neurologic sequelae. Clearly, additional studies are needed to determine optimal antiparasitic and anti-inflammatory therapy for this severe form of disease.

## References

[1] White AC Coyle CM Rajshekhar V Singh G Hauser WA Mohanty A Garcia HH Nash TE, 2018 Diagnosis and treatment of neurocysticercosis: 2017 clinical practice guidelines by the Infectious Diseases Society of America (IDSA) and the American Society of Tropical medicine and Hygiene (ASTMH). Am J Trop Med Hyg 98: 945–966. 29644966 10.4269/ajtmh.18-88751 PMC5928844

[2] Garcia HH 2014 Efficacy of combined antiparasitic therapy with praziquantel and albendazole for neurocysticercosis: a double-blind, randomised controlled trial. Lancet Infect Dis 14: 687–695. 24999157 10.1016/S1473-3099(14)70779-0 PMC4157934

[3] Moyano LM 2016 High prevalence of asymptomatic neurocysticercosis in an endemic rural community in Peru. PLoS Negl Trop Dis 10: e0005130. 27992429 10.1371/journal.pntd.0005130 PMC5167259

[4] Del Brutto OH Arroyo G Del Brutto VJ Zambrano M García HH, 2017 On the relationship between calcified neurocysticercosis and epilepsy in an endemic village: a large-scale, computed tomography-based population study in rural Ecuador. Epilepsia 58: 1955–1961. 28850668 10.1111/epi.13892 PMC5670011

[5] Fleury A 2003 High prevalence of calcified silent neurocysticercosis in a rural village of Mexico. Neuroepidemiology 22: 139–145. 12629280 10.1159/000068748

[6] Marcin Sierra M 2017 Extraparenchymal neurocysticercosis: demographic, clinicoradiological, and inflammatory features. PLoS Negl Trop Dis 11: e0005646. 28599004 10.1371/journal.pntd.0005646 PMC5479594

[7] Sotelo J Marin C, 1987 Hydrocephalus secondary to cysticercotic arachnoiditis. A long-term follow-up review of 92 cases. J Neurosurg 66: 686–689. 3572494 10.3171/jns.1987.66.5.0686

[8] Sorvillo FJ DeGiorgio C Waterman SH, 2007 Deaths from cysticercosis, United States. Emerg Infect Dis 13: 230–235. 17479884 10.3201/eid1302.060527 PMC2725874

[9] Nash T O’Connell E Hammoud D Wetzler L Ware J Mahanty S, 2020 Natural history of treated subarachnoid racemose neurocysticercosis. Am J Trop Med Hyg 102: 78–89. 31642423 10.4269/ajtmh.19-0436 PMC6947806

[10] Osorio R Carrillo-Mezo R Romo ML Toledo A Matus C González-Hernández I Jung H Fleury A, 2019 Factors associated with cysticidal treatment response in extraparenchymal neurocysticercosis. J Clin Pharmacol 59: 548–556. 30476351 10.1002/jcph.1346

[11] Serpa JA Graviss EA Kass JS White AC, 2011 Neurocysticercosis in Houston, Texas: an update. Medicine (Baltimore) 90: 81–86. 21200189 10.1097/MD.0b013e318206d13e

[12] Sotelo J Escobedo F Rodriguez-Carbajal J Torres B Rubio-Donnadieu F, 1984 Therapy of parenchymal brain cysticercosis with praziquantel. N Engl J Med 310: 1001–1007. 6708975 10.1056/NEJM198404193101601

[13] Escobedo F Penagos P Rodriguez J Sotelo J, 1987 Albendazole therapy for neurocysticercosis. Arch Intern Med 147: 738–741. 3827462

[14] Carpio A Santillán F León P Flores C Hauser WA, 1995 Is the course of neurocysticercosis modified by treatment with antihelminthic agents? Arch Intern Med 155: 1982–1988. 7575052

[15] Garcia HH Pretell EJ Gilman RH Martinez SM Moulton LH Del Brutto OH Herrera G Evans CA Gonzalez AE; Cysticercosis Working Group in Peru, 2004 A trial of antiparasitic treatment to reduce the rate of seizures due to cerebral cysticercosis. N Engl J Med 350: 249–258. 14724304 10.1056/NEJMoa031294

[16] Carpio A Kelvin E Bagiella E Leslie D Leon P Andrews H Hauser W, 2008 Effects of albendazole treatment on neurocysticercosis: a randomised controlled trial. J Neurol Neurosurg Psychiatry 79: 1050–1055. 18495737 10.1136/jnnp.2008.144899

[17] Zhao BC 2016 Albendazole and corticosteroids for the treatment of solitary cysticercus granuloma: a network meta-analysis. PLoS Negl Trop Dis 10: e0004418. 26849048 10.1371/journal.pntd.0004418 PMC4744042

[18] Agapejev S Da Silva MD Ueda AK, 1996 Severe forms of neurocysticercosis: treatment with albendazole. Arq Neuropsiquiatr 54: 82–93. 8736150 10.1590/s0004-282x1996000100014

[19] Proaño JV Madrazo I Avelar F Lopez-Felix B Diaz G Grijalva I, 2001 Medical treatment for neurocysticercosis characterized by giant subarachnoid cysts. N Engl J Med 345: 879–885. 11565520 10.1056/NEJMoa010212

[20] Nash TE Ware JM Coyle CM Mahanty S, 2019 Etanercept to control inflammation in the treatment of complicated neurocysticercosis. Am J Trop Med Hyg 100: 609–616. 30608049 10.4269/ajtmh.18-0795 PMC6402894

[21] Kelley R Duong DH Locke GE, 2002 Characteristics of ventricular shunt malfunctions among patients with neurocysticercosis. Neurosurgery 50: 757–761; discussion 761–762. 11904026 10.1097/00006123-200204000-00014

[22] Suastegui Roman RA Soto-Hernandez JL Sotelo J, 1996 Effects of prednisone on ventriculoperitoneal shunt function in hydrocephalus secondary to cysticercosis: a preliminary study. J Neurosurg 84: 629–633. 8613855 10.3171/jns.1996.84.4.0629

[23] Proaño JV Torres-Corzo J Rodríguez-Della Vecchia R Guizar-Sahagun G Rangel-Castilla L, 2009 Intraventricular and subarachnoid basal cisterns neurocysticercosis: a comparative study between traditional treatment versus neuroendoscopic surgery. Childs Nerv Syst 25: 1467–1475. 19557421 10.1007/s00381-009-0933-4

[24] Fleury A Garcia E Hernández M Carrillo R Govezensky T Fragoso G Sciutto E Harrison LJ Parkhouse RM, 2013 Neurocysticercosis: HP10 antigen detection is useful for the follow-up of the severe patients. PLoS Negl Trop Dis 7: e2096. 23505587 10.1371/journal.pntd.0002096 PMC3591315

[25] O’Connell EM Harrison S Dahlstrom E Nash T Nutman TB, 2019 A novel, highly sensitive qPCR assay for the diagnosis of subarachnoid and ventricular neurocysticercosis and for assessing response to treatment. Clin Infect Dis. Available at https//.org/10.1093/cid/ciz541. 10.1093/cid/ciz541 PMC7156770 31232448

